# Auditory complications among childhood cancer survivors and health-related quality of life: a PanCareLIFE study

**DOI:** 10.1007/s11764-023-01456-4

**Published:** 2023-09-22

**Authors:** Sven Strebel, Katja Baust, Desiree Grabow, Julianne Byrne, Thorsten Langer, Antoinette am Zehnhoff-Dinnesen, Rahel Kuonen, Annette Weiss, Tomas Kepak, Jarmila Kruseova, Claire Berger, Gabriele Calaminus, Grit Sommer, Claudia E. Kuehni, on behalf of the PanCareLIFE Consortium

**Affiliations:** 1 Institute of Social and Preventive Medicine, University of Bern, Mittelstrasse 43, 3012 Bern, Switzerland; 2 CANSEARCH Research Platform in Pediatric Oncology and Hematology, Department of Pediatrics, Gynecology and Obstetrics, University of Geneva, Geneva, Switzerland; 3 Graduate School for Health Sciences, University of Bern, Bern, Switzerland; 4 Department of Pediatric Hematology and Oncology, University Hospital Bonn, Bonn, Germany; 5 Division of Childhood Cancer Epidemiology, German Childhood Cancer Registry, Institute of Medical Biostatistics, Epidemiology and Informatics (IMBEI), University Medical Center of the Johannes Gutenberg University Mainz, Mainz, Germany; 6 Boyne Research Institute, Drogheda, Ireland; 7 Department of Pediatric Oncology and Hematology, University Hospital for Children and Adolescents, Lübeck, Germany; 8 Department for Phoniatrics and Pedaudiology, University Hospital Münster, Westphalian Wilhems University, Münster, Germany; 9 Department of Pediatrics, Inselspital, Bern University Hospital, University of Bern, Bern, Switzerland; 10 Bavarian Care and Nursing Authority, Amberg, Germany; 11 University Hospital Brno & International Clinical Research Center (FNUSA-ICRC), Masaryk University, Brno, Czech Republic; 12 Department of Pediatric Hematology and Oncology, 2nd Faculty of Medicine, Charles University and University Hospital Motol, Prague, Czech Republic; 13 Department of Pediatric Hematology and Oncology, University-Hospital, Saint-Étienne, France; 14 Lyon University, Jean Monnet University, INSERM U 1059, Sainbiose, Saint-Étienne, France; 15 Department of Pediatric Endocrinology, Diabetology and Metabolism, Inselspital, Bern University Children’s Hospital, University of Bern, Bern, Switzerland; 16 Department of BioMedical Research, University of Bern, Bern, Switzerland; 17 Division of Pediatric Hematology/Oncology, Department of Pediatrics, Inselspital, Bern University Hospital, University of Bern, Bern, Switzerland

**Keywords:** Childhood cancer, Survivorship, Quality of life, Hearing loss, Tinnitus

## Abstract

**Purpose:**

Auditory complications are potential side effects from childhood cancer treatment. Yet, limited evidence exists about the impact of auditory complications—particularly tinnitus—on health-related quality of life (HRQoL) among childhood cancer survivors (CCS). We determined the prevalence of hearing loss and tinnitus in the European PanCareLIFE cohort of CCS and examined its effect on HRQoL.

**Methods:**

We included CCS from four European countries who were diagnosed at age ≤ 18 years; survived ≥ 5 years; and aged 25–44 years at study. We assessed HRQoL (Short Form 36), hearing loss, and tinnitus using questionnaires. We used multivariable linear regression to examine associations between these two auditory complications and HRQoL adjusting for socio-demographic and clinical factors.

**Results:**

Our study population consisted of 6,318 CCS (53% female; median age at cancer diagnosis 9 years interquartile range [IQR] 5–13 years) with median age at survey of 31 years (IQR 28–35 years). Prevalence was 7.5% (476/6,318; confidence interval [CI]: 6.9–8.2) for hearing loss and 7.6% (127/1,668; CI: 6.4–9.0) for tinnitus. CCS with hearing loss had impaired physical (coefficient [coef.] -4.3, CI: -7.0 to -1.6) and mental (coef. -3.2, CI: -5.5 to -0.8) HRQoL when compared with CCS with normal hearing. Tinnitus was associated with impaired physical (coef. -8.2, CI: -11.8 to -4.7) and mental (coef. -5.9, CI: -8.8 to -3.1) HRQoL.

**Conclusion:**

We observed reduced HRQoL among CCS with hearing loss and tinnitus. Our findings indicate timely treatment of hearing loss and tinnitus may contribute to quality of life of survivors.

**Implications for cancer survivors:**

CCS who experience auditory complications should be counseled about possible therapeutic and supportive measures during follow-up care.

**Supplementary information:**

The online version contains supplementary material available at 10.1007/s11764-023-01456-4.

## Introduction

Cancer treatment can cause auditory complications, such as hearing loss and tinnitus [1, 2]. In recent surveys, childhood cancer survivors (CCS) reported more hearing loss and tinnitus when compared with their siblings [3, 4]. Ototoxic cancer treatments include platinum-based chemotherapy, cranial radiotherapy (CRT), and surgeries involving the auditory system [1, 4, 5]. Other suspected ototoxic treatments are concomitant medications such as aminoglycosides or loop diuretics, hematopoietic stem cell transplantation (HSCT), or the neurotoxic vinca alkaloid vincristine [5–7]. Hearing loss and tinnitus lead to a wide range of educational and psychosocial problems such as learning difficulties and emotional distress among CCS and the general population [8–11]. The overall burden of auditory complications ultimately affects health-related quality of life (HRQoL) of CCS [12–14]. However, there is still a lack of awareness of therapeutic options, especially regarding the treatment of tinnitus [15].

Only a few studies have investigated auditory complications and how they affect HRQoL among CCS [12–14]. Previous studies with small sample sizes and heterogeneous inclusion criteria make comparisons between studies difficult since findings can only be extrapolated to the overall CCS population to a limited extent. The association of tinnitus with HRQoL among CCS remains unknown. Several studies examined the prevalence of hearing loss among CCS treated with cisplatin or CRT, yet studies of the overall population—and studies investigating tinnitus—are scarce [3, 4, 16, 17]. We thus combined harmonized data from four European countries into a large cohort of CCS to describe the prevalence of hearing loss and tinnitus and investigate their association with HRQoL.

## Methods

### Study population

PanCareLIFE (PCL) is a European-based study on late effects among CCS [18]. It focuses on hearing loss, fertility problems, and quality of life [19, 20]. For the current study, we included CCS from Switzerland (CH), Czech Republic (CZ), Germany (DE), and France (FR). For the cohort from the Netherlands, data on hearing loss or tinnitus were not available within the PCL data set. The study population included national or regional cohorts of CCS (1) diagnosed with cancer according to the International Classification of Childhood Cancer (ICCC-3), 3rd edition [21], or Langerhans cell histiocytosis; (2) aged ≤ 18 years at time of cancer diagnosis; (3) survived ≥ 5 years after cancer diagnosis; (4) were off treatment for cancer at time of study; (5) aged 25–44 years when they received the questionnaire. To make data comparable between countries, we restricted our analysis to CCS ≥ 25 years and < 45 years because data for CCS younger than 25 years were unavailable for the German cohort and data for CCS older than 45 years were unavailable for the French cohort. Details about study design, recruitment of participants, country-specific exclusion criteria, characteristics of different cohorts, and a non-responder analysis were published in a separate study protocol [19].

### Study procedure

Each country sent questionnaires to their respective regional or national cohorts between 2005 and 2017 [19]. The questionnaires were sent by mail except in CZ where clinic staff distributed them during follow-up visits to former patients. The questionnaire included questions about HRQoL, hearing, socio-demographic characteristics, and lifestyle behavior. Non-responders were reminded to complete the questionnaire [19]. Clinical information on cancer diagnosis and treatment was extracted from medical records by each participating country.

### Assessment of HRQoL

We assessed HRQoL with the Short-Form 36 (SF-36) questionnaire [22]. The SF-36 is a widely used instrument; several studies used it to determine HRQoL among CCS [12, 23–26]. The questionnaire includes 36 items covering different aspects of physical and mental health aggregated into eight health domains: physical functioning (PF, 10 items), role-limitations due to physical problems (RP, 4 items), bodily pain (BP, 2 items), general health (GH, 5 items), vitality (VT, 4 items), social functioning (SF, 2 items), role-limitations due to emotional problems (RE, 3 items) and mental health (MH, 5 items) [22, 27]. These health domains are further collapsed into summary scores that reflect overall physical and mental health: physical component summary (PCS) and mental component summary (MCS). We converted all raw scores into T-scores ranging from 0 to 100 for each health domain. A higher score indicates better HRQoL. The T-scores were further transformed according to reference data from the German norm population stratified for age and sex (mean = 50, SD = 10) [19, 28].

### Auditory complications

We defined self-reported hearing loss (yes, no) and tinnitus (yes, no) as our main determinants of interest for impaired HRQoL. Participating country questionnaires contained slightly differently worded questions on hearing (Supplement Table S1). The central PCL data center in Mainz (Germany) aggregated data and harmonized variables between participating countries in 2017 [19]. Data on tinnitus (yes, no) were unavailable for the German cohort; thus, we excluded German data for analyses involving tinnitus. We coded missing answers for hearing loss (< 1%) and tinnitus (5%) as normal hearing and without tinnitus. We assumed that CCS with hearing loss or tinnitus would be more likely to answer the question than CCS without auditory complications.

### Clinical and socio-demographic information

Based on previous study findings, we collected clinical and socio-demographic factors possibly associated with HRQoL among CCS: sex (female, male); age at survey; migration background (yes, no); education (primary, secondary, tertiary); occupational status (employed, unemployed); living with a partner (yes, no); currently smoking tobacco (yes, no); drinking $>$1 alcoholic beverage per week (yes, no); body mass index (BMI); cancer diagnosis according to ICCC-3 [21]; age at diagnosis; history of relapse (yes, no); surgery (yes, no); chemotherapy (yes, no); radiotherapy (yes, no); HSCT (yes, no) [12, 23, 25, 29]. Respondents self-reported age at survey, migration background, education, occupational status, living with a partner, tobacco smoking status, alcohol consumption, and BMI variables [19]. Demographic, cancer-related, and treatment information were extracted from participating institution medical records or corresponding cancer registries [19].

### Statistical analysis

We used *t*-tests and fitted multivariable linear regression models to investigate possible associations of hearing loss or tinnitus with HRQoL. First, we examined whether mean scores on SF-36 health domains and PCS and MCS scores differed between CCS with and without auditory complications. We then fitted multivariable linear regression models to investigate whether any possible association of hearing loss or tinnitus with health domains and PCS and MCS scores were explained by clinical or socio-demographic factors. We chose linear regression because HRQoL outcome variables are continuous and binary categorizations of HRQoL measured by SF-36 is without consensus in the literature. To mitigate effects of sample imbalances between countries, we standardized cohorts from CZ, DE, and FR according to age at survey and sex variables. Because of the balanced distribution across all age groups and genders, we used the CH cohort as the reference population to calculate appropriate weights. Based on the conceptual framework of directed acyclic graphs (Supplement Fig. S1) [30, 31], we adjusted our models for the following co-variables: age at survey (continuous in years); age at cancer diagnosis (continuous in years); type of cancer (categorical according ICCC-3); history of relapse (yes, no); surgery (yes, no); chemotherapy (yes, no); radiotherapy (yes, no); and HSCT (yes, no). We decided to include country of data provider to adjust for country-specific differences in recruitment of study participants and audiological monitoring [19, 32, 33]. We calculated global *p*-values using the Wald test.

Since we hypothesized that the burden of auditory complications may be greatest among CCS with both tinnitus and hearing loss [34], following the suggestion that strength of effect and dose-response support a causal relationship [35], we performed a sub-analysis to further investigate a potential causal relationship. There, we coded auditory complications as either (1) no auditory complications; (2) hearing loss only; (3) tinnitus only; or (4) hearing loss and tinnitus.

We used Stata version 16.1 (StataCorp LP, Austin, Texas) for all analyses. For the creation of the directed acyclic graph we used the R package ‘dagitty’.

## Results

### Characteristics of study population

In total, 6,318 CCS were available for our analysis. Of the 6,318 CCS, most were from DE (*n* = 4,650; 74%); 822 (13%) from CH; 592 (9%) from CZ; and 254 (4%) from FR (Table 1). Our study population included 3,326 (53%) females and 2,992 (47%) males with median age of 31 (interquartile range [IQR] 28–35 years) at survey, median age 9 (IQR 5–13 years) at cancer diagnosis, and median 23 years (IQR 19–28) since cancer diagnosis. Leukemias (*n* = 2,033; 32%), lymphomas (*n* = 1,466; 23%), and central nervous system (CNS) tumors (*n* = 892; 14%) were the most common cancer diagnoses. CCS received cancer treatment by surgery only (258; 4%); chemotherapy only (1,099; 17%); radiotherapy only (22; <1%); surgery and chemotherapy (827; 13%); surgery and radiotherapy (129; 2%); radiotherapy and chemotherapy (1,493; 24%); surgery, chemotherapy, and radiotherapy (1,174; 19%); no surgery, chemotherapy, or radiotherapy (12; <1%); and HSCT (139; 2%). For 1,165 (18%) of CCS complete treatment information was not available.

**Table 1:** Demographic and clinical characteristics of study population

	Total cohort	
	(*N* = 6,318)	
Demographic characteristics	*n*	(%)
Sex		
Male	2,992	(47)
Female	3,326	(53)
Age at survey (years)		
25-29.9	2,651	(42)
30-34.9	2,061	(33)
35-39.9	1,074	(17)
40–44	532	(8)
Country of origin		
Germany	4,650	(74)
Switzerland	822	(13)
Czech Republic	592	(9)
France	254	(4)
Clinical characteristics	*n*	(%)
Age at cancer diagnosis (years)		
0-4.9	1,763	(28)
5-9.9	1,696	(27)
10–18	2,859	(45)
Period of cancer diagnosis		
1974–1984	1,254	(20)
1985–1994	3,488	(55)
1995–2004	1,517	(24)
2005–2009	59	(1)
Time since cancer diagnosis (years)		
5-14.9	592	(9)
15-24.9	3,208	(51)
25–42	2,518	(40)
Cancer diagnosis (ICCC-3)		
I Leukemias	2,033	(32)
II Lymphomas	1,466	(23)
III CNS tumours	892	(14)
IV Neuroblastoma	236	(4)
V Retinoblastoma	130	(2)
VI Renal tumours	390	(6)
VII Hepatic tumours	28	(< 1)
VIII Bone tumours	378	(6)
IX Soft tissue sarcomas	396	(6)
X Germ cell tumours	249	(4)
XI Epithelial neoplasms & melanomas	80	(1)
XII Other malignant neoplasms	40	(1)
Subsequent tumour		
Yes	458	(7)
No	5,860	(93)
Treatments ^a^		
Surgery		
Unknown	863	(14)
Yes	2,580	(41)
No	2,875	(46)
Chemotherapy		
Unknown	603	(10)
Yes	5,070	(80)
No	645	(10)
Radiotherapy		
Unknown	858	(14)
Yes	3,092	(49)
No	2,368	(37)
HSCT		
Unknown	287	(5)
Yes	151	(2)
No	5,880	(93)

Abbreviations: ICCC-3, International Classification of Childhood Cancer–Third Edition; CNS, central nervous system; HSCT, hematopoietic stem cell transplantation

^a^Each subject could have had more than one treatment modality

### Prevalence of auditory complications after childhood cancer

**Fig. 1:**
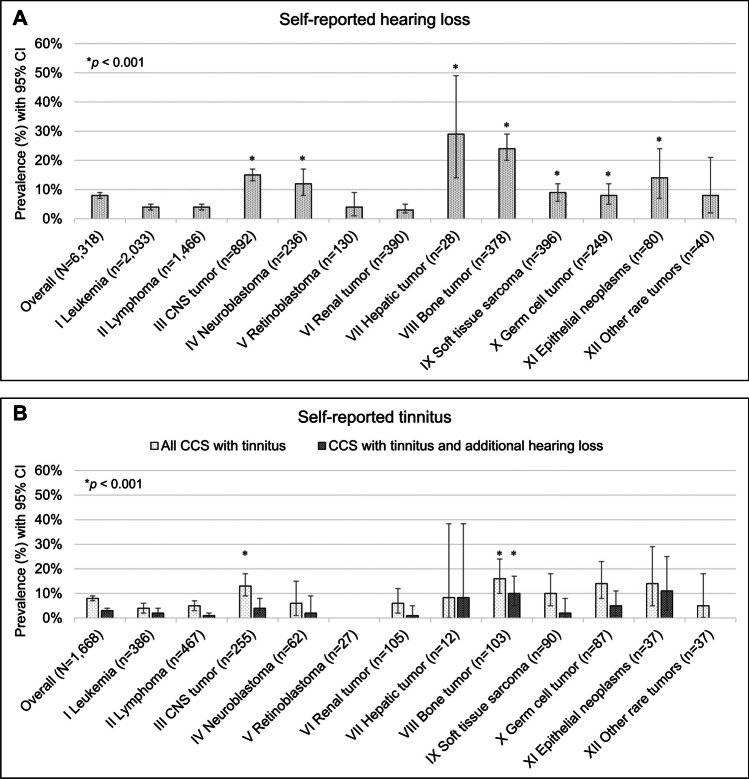
Prevalence of self-reported auditory complications at the time of the study. *P*-values are calculated from chi^2^-statistics comparing prevalence between survivors of leukemia with survivors of other tumor types. **A** Prevalence of self-reported hearing loss (*N* = 6,318). **B** Prevalence of self-reported tinnitus (*N* = 1,668), including data from Switzerland, Czech Republic and France. No data on tinnitus was available for the German cohort (*n* = 4,650). Abbreviations: CNS, central nervous system

Of participating CCS, 7.5% (476/6,318; CI: 6.9–8.2) reported hearing loss. Data on tinnitus was available for the cohorts from CH, CZ, and FR resulting in a combined cohort of 1,668 CCS. Of those, 7.6% (127/1,668; CI: 6.4-9.0) reported tinnitus. Among CCS with tinnitus (*n* = 127), 45 (35%) also reported hearing loss. CCS diagnosed with CNS tumors, neuroblastoma, hepatic tumors, malignant bone tumors, soft tissue sarcomas, germ cell tumors, and epithelial neoplasms reported hearing loss more often than CCS diagnosed with leukemia (all *p* < 0.001) (Fig. 1A). CCS of hepatic tumors had the highest prevalence of hearing loss (8/28; 28.6%, CI: 13.2–48.7) followed by malignant bone tumors (91/378; 24.1%, CI: 19.8–28.7) and CNS tumors (130/892; 14.6%, CI: 12.3–17.1). Tinnitus prevalence was highest among CCS diagnosed with malignant bone tumors (16/103; 15.5%, CI: 9.1–24.0) and CNS tumors (33/255; 12.9%, CI: 9.1–17.7) (Fig. 1B).

### Association of auditory complications with HRQoL

**Fig. 2:**
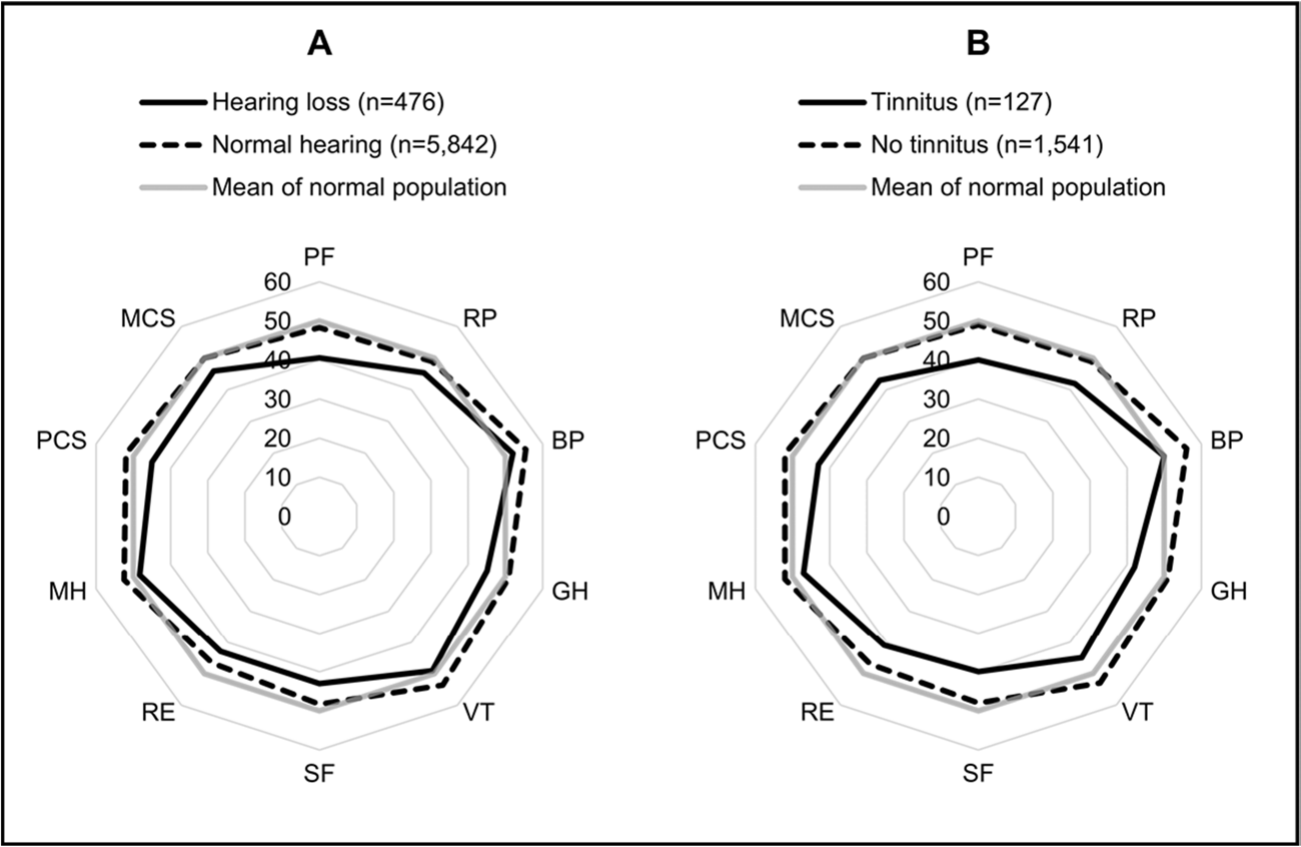
The two spider charts (**A**, **B**) show norm-based mean scores for all eight health domains and the two summary scores of the SF-36 comparing (**A**) CCS with hearing loss and normal hearing (*N* = 6318) and (**B**) CCS with tinnitus only, tinnitus and hearing loss, and without tinnitus (*N* = 1668). We included data from Switzerland, Czech Republic and France for the analysis of the association of tinnitus on HRQoL (**B**) (*N* = 1668). No data on tinnitus was available for the German cohort (*n* = 4650). Higher scores indicate better HRQoL. Normal population (grey line) has an estimated mean score of 50 with a standard deviation of 10 for all HRQoL scores of the SF-36. Raw data of the figure are shown in the supplement (Supplement Tables S2, S3). Abbreviations: *PF *physical functioning, *RP *role physical, *BP *bodily pain, *GH *general health, *VT *vitality, *SF *social functioning, *RE *role emotional, *MH *mental health, *PCS *physical component summary, *MCS *mental component summary

 CCS with hearing loss had lower HRQoL mean scores than CCS with normal hearing (all differences with *p* < 0.001) (Fig. 2A). Looking at SF-36 summary scores, CCS with hearing loss scored 45.3 in overall physical (PCS) and 46.0 in overall mental (MCS) HRQoL. In comparison, CCS with normal hearing had 51.7 for PCS and 50.0 for MCS scores. Among the eight health domains, we observed the largest mean differences between CCS with and without hearing loss in physical functioning (40.6 vs. 48.3), general health (45.1 vs. 50.9), and social functioning (43.0 vs. 48.2).

CCS with tinnitus scored lower than CCS without tinnitus in all health domains and PCS (42.7 vs. 52.2) and MCS (43.1 vs. 49.9) summary scores (all differences with *p* < 0.001) (Fig. 2B). The largest mean differences between CCS with and without tinnitus were again in physical functioning (40.2 vs. 48.9), general health (41.6 vs. 51.0), and social functioning (39.7 vs. 48.3).

In the multivariable linear regression, hearing loss remained associated with lower HRQoL scores after adjusting for socio-demographic and cancer-related factors (Table 2). For PCS, coef. were -4.3 (CI: -7.0 to -1.6) among those with hearing loss and -3.2 (CI: -5.5 to -0.8) for MCS. On average, overall physical or mental HRQoL was reduced by 4.3 or 3.2 points for CCS with hearing loss compared with CCS with normal hearing. The association was strongest for general health, followed by physical functioning, vitality, and social functioning (coef. ranging from -4.6 to -3.8, *p* < 0.05) (Table 3). We observed borderline or no associations of hearing loss in role physical and role emotional.

**Table 2:** Association of hearing loss and tinnitus with HRQoL from adjusted linear regression analysis

	Adjusted^a^ association of hearing loss^b^		Adjusted^a^ association of tinnitus^c^	
Health Domains	Coef. (95% CI)	*P*^d^	Coef. (95% CI)	*P*^d^
Physical Functioning (PF)	-4.5 (-7.7 to -1.4)	0.005	-7.2 (-11.2 to -3.2)	< 0.001
Role Physical (RP)	-2.3 (-4.6 to -0.1)	0.044	-5.9 (-8.8 to -2.9)	< 0.001
Role Emotional (RE)	-2.5 (-5.4 to 0.4)	0.088	-3.9 (-7.0 to -0.8)	0.014
Bodily Pain (BP)	-2.6 (-4.4 to -0.8)	0.004	-5.3 (-7.6 to -3.0)	< 0.001
Mental Health (MH)	-2.8 (-4.7 to -0.8)	0.005	-4.2 (-6.9 to -1.6)	0.002
Vitality (VT)	-3.9 (-6.0 to -1.8)	< 0.001	-8.4 (-11.3 to -5.6)	< 0.001
General Health (GH)	-4.6 (-6.9 to -2.3)	< 0.001	-8.3 (-11.4 to -5.3)	< 0.001
Social Functioning (SF)	-3.8 (-6.3 to -1.3)	0.003	-7.9 (-11.0 to -4.7)	< 0.001
Global Summary Scores	Coef. (95% CI)	*P*^d^	Coef. (95% CI)	*P*^d^
PCS	-4.3 (-7.0 to -1.6)	0.002	-8.2 (-11.8 to -4.7)	< 0.001
MCS	-3.2 (-5.5 to -0.8)	0.008	-5.9 (-8.8 to -3.1)	< 0.001

Abbreviations: Coef., estimated beta coefficient from multivariable linear regression; PCS, physical component summary; MCS, mental component summary

^a^Adjusted for: age at survey (continuous, in years); age at cancer diagnosis (continuous, in years); type of cancer diagnosis (according ICCC-3); history of relapse (yes, no); surgery (yes, no); chemotherapy (yes, no); radiotherapy (yes, no); HSCT (yes, no); country of data provider

^b^We included the total cohort for analysis of the association of hearing loss on HRQoL (*N* = 6,318)

^c^We included data from Switzerland, Czech Republic and France for the analysis of the association of tinnitus on HRQoL (*N* = 1,668). No data on tinnitus was available for the German cohort (*n* = 4,650)

^d^P-value calculated from Wald test

Tinnitus also remained associated with lower HRQoL scores in multivariable linear regression (Table 2). The effects of tinnitus on PCS (coef. -8.2, CI: -11.8 to -4.7) and MCS (coef. -5.9, CI: -8.8 to -3.1) were greater compared with the effects of hearing loss (coef. of -4.3 for PCS and -3.2 for MCS). We found the strongest effect of tinnitus on vitality (coef. -8.4, CI: -11.3 to -5.6), general health (coef. -8.3, CI: -11.4 to -5.3), and social functioning (coef. -7.9, CI: -11.0 to -4.7) (all *p* < 0.001).

We found CCS with both tinnitus and hearing loss had lower overall physical and mental HRQoL compared with CCS with hearing loss alone (coef. -14.5 vs. -0.6 for PCS and coef. -5.0 vs. -2.9 for MCS) (Table 3). When compared with tinnitus alone, the effect of hearing loss and additional tinnitus was also larger for overall physical HRQoL (coef. -5.4 vs. -14.5 for PCS), yet similar for overall mental HRQoL (coef. -6.8 vs. -5.0 for MCS).

**Table 3:** Association of combined auditory complications on HRQoL from adjusted linear regression analysis

	Adjusted^a^ association of combined auditory complications	
Health Domains	Coef. (95% CI)	*P*^c^
Physical Functioning (PF)		0.002
No auditory complications	*Reference*	
Hearing loss only	-0.1 (-3.3 to 3.1)	
Tinnitus only	-3.3 (-6.6 to 0.1)	
Hearing loss and tinnitus	-15.5 (-24.8 to -6.3)	
Role Physical (RP)		0.002
No auditory complications	*Reference*	
Hearing loss only	0.1 (-2.5 to 2.7)	
Tinnitus only	-4.5 (-7.5 to -1.4)	
Hearing loss and tinnitus	-8.8 (-15.2 to -2.5)	
Role Emotional (RE)		0.053
No auditory complications	*Reference*	
Hearing loss only	-2.0 (-6.2 to 2.2)	
Tinnitus only	-4.0 (-7.2 to -0.8)	
Hearing loss and tinnitus	-4.3 (-11.0 to 2.3)	
Bodily Pain (BP)		< 0.001
No auditory complications	*Reference*	
Hearing loss only	-1.1 (-3.5 to 1.2)	
Tinnitus only	-4.4 (-7.0 to -1.7)	
Hearing loss and tinnitus	-7.6 (-11.7 to -3.5)	
Mental Health (MH)		0.007
No auditory complications	*Reference*	
Hearing loss only	-2.3 (-5.1 to 0.6)	
Tinnitus only	-4.5 (-7.8 to -1.1)	
Hearing loss and tinnitus	-4.3 (-8.3 to -0.3)	
Vitality (VT)		< 0.001
No auditory complications	*Reference*	
Hearing loss only	-3.0 (-6.1 to 0.1)	
Tinnitus only	-8.7 (-12.2 to -5.2)	
Hearing loss and tinnitus	-8.6 (-13.1 to -4.2)	
General Health (GH)		< 0.001
No auditory complications	*Reference*	
Hearing loss only	-2.6 (-6.1 to 0.9)	
Tinnitus only	-7.2 (-11.0 to -3.4)	
Hearing loss and tinnitus	-11.4 (-15.8 to -7.0)	
Social Functioning (SF)		< 0.001
No auditory complications	*Reference*	
Hearing loss only	-1.6 (-5.0 to 1.8)	
Tinnitus only	-7.0 (-10.4 to -3.6)	
Hearing loss and tinnitus	-10.0 (-16.2 to -3.9)	
Global Summary Scores	Coef. (95% CI)	*P*^c^
PCS		< 0.001
No auditory complications	*Reference*	
Hearing loss only	-0.6 (-3.5 to 2.2)	
Tinnitus only	-5.4 (-8.8 to -1.9)	
Hearing loss and tinnitus	-14.5 (-21.9 to -7.1)	
MCS		< 0.001
No auditory complications	*Reference*	
Hearing loss only	-2.9 (-6.2 to 0.5)	
Tinnitus only	-6.8 (-10.1 to -3.5)	
Hearing loss and tinnitus	-5.0 (-10.0 to 0.1)	

Abbreviations: PCS, physical component summary; MCS, mental component summary

^a^Adjusted for: age at survey (continuous, in years); age at cancer diagnosis (continuous, in years); type of cancer diagnosis (according ICCC-3); history of relapse (yes, no); surgery (yes, no); chemotherapy (yes, no); radiotherapy (yes, no); HSCT (yes, no); country of data provider

^b^We included the cohorts from CH, CZ, and FR for the analysis (*N* = 1,668) but excluded the cohort from Germany (*n* = 4,650) because no data on tinnitus was available for the German cohort

^c^*P*-value calculated from Wald test

## Discussion

We found the prevalence of auditory complications varied between cancer diagnoses and the highest prevalence of hearing loss and tinnitus among survivors of CNS and malignant bone tumors. HRQoL was lower among CCS with auditory complications than for those with normal hearing. Hearing loss and tinnitus were strongly associated with physical functioning, vitality, general health, and social functioning. We observed lower HRQoL among CCS with hearing loss and additional tinnitus compared with CCS with hearing loss alone.

### Strengths and limitations

Since our study is the largest cohort of CCS to examine auditory complications and their association with HRQoL, it results in high statistical power and good representativeness because it combined data from population-based and regional well-defined cohorts. Tinnitus is more frequent among CCS compared with the general population [2], yet its association with HRQoL among CCS was unknown. We included CCS with all possible cancer treatments, not only those exposed to ototoxic treatments such as platinum-based chemotherapy or CRT, which allowed assessing the burden of auditory complications among the overall population of CCS [36]. We used self-reported data on hearing loss and tinnitus, which agree well with audiograms from medical reports, although they underestimate mild and unilateral hearing loss [37]. To assess the impact of auditory complications on quality of life, self-reported data on hearing might be more appropriate than audiograms, because it directly reflect the survivors’ experience. Asymptomatic high-frequency hearing loss that is detected only through an audiogram but is not apparent to the survivor may not affect HRQOL. We applied SF-36—an established and validated instrument widely used in previous studies—to measure HRQoL among CCS, which allows comparing our data with other studies [23–25, 29, 38, 39]. Data from participating countries were collected centrally and harmonized before merging to avoid data management errors [19]. However, our study results might still be influenced by study design differences of participating countries, leading to potential selection bias. For example, FR did not contact CCS of leukemias, and—similar to DE—sent questionnaires later (≥ 10 years) than CH and CZ (both ≥ 5 years) after cancer diagnosis [19]. Considering the French cohort represents only 4% of the total study population, we assume that selection based on cancer diagnosis did not result in a major bias in our findings. Additionally, time since diagnosis was investigated in two larger population-based studies showing either no or minor effects on HRQoL among CCS [25, 29]. Other limitations relate to the main exposures of interest; hearing loss and tinnitus. Since auditory complications were assessed by questionnaire and dependent on severity, underreporting is possible. For instance, CCS with severe hearing loss possibly received better audiologic care and recall it better than CCS with mild high-frequency hearing loss who are unaware of it [37]. PCL is a large collaborative research project across multiple countries and cohorts examining various late effects and their impact on quality of life. For this reason, we chose the SF-36 questionnaire as an established and validated instrument to measure different aspects of HRQoL in CCS. However, the SF-36 does not specifically measure HRQoL related to hearing and may not capture all life situations affected by auditory complications. In addition, any observed correlation between auditory complications and decreased HRQoL must be interpreted with caution because the more general domains of HRQoL measured by the SF-36 may also be affected by other late effects.

### Comparison with previous studies

Among our study population, 7.5% of CCS reported hearing loss. We observed particularly high prevalence among survivors of hepatoblastoma, CNS tumors, and malignant bone tumors—an expected finding from higher cisplatin or CRT use compared with other cancer treatment regimes [5, 7]. Larger studies on hearing loss prevalence among CCS mostly focused on high-risk populations treated with platinum-based chemotherapy or CRT [5–7]. Two population-based studies from Switzerland (Swiss Childhood Cancer Survivor Study; SCCSS) and the United States (Childhood Cancer Survivor Study; CCSS) determined the prevalence of hearing loss among the overall CCS population with questionnaires [3, 17]. The Swiss population in our cohort overlaps with the study population of the SCCSS [3, 19, 40]. Therefore, we only compared our data with the CCSS study [17]. Whelan and colleagues found a prevalence of self-reported hearing loss of 5%, which is slightly lower than what we found (7.5%) [17]. They included CCS diagnosed in earlier years (1970–1986) compared with our study (1974–2009), which possibly explains the difference. Considering cisplatin was first approved in 1978 for adult cancer treatment, it is possible a higher proportion of CCS in our cohort were treated with ototoxic platinum-based chemotherapy, as Weiss and colleagues also discuss in their SCCSS study [3, 41].

The prevalence of tinnitus was 7.6% among our study population. CCS diagnosed with CNS tumors or malignant bone tumors had a three to four times higher prevalence compared with survivors of leukemias. The higher prevalence is possibly explained by previously identified risk factors for tinnitus among CCS, such as exposure to cisplatin, CRT, and CNS surgeries [4]. In Meijer and colleagues’ systematic review, the prevalence of tinnitus ranged from 3 to 17% [2]. They also recently published a population-based study where they estimated the prevalence of tinnitus to be 9.5% among CCS compared with 3.7% for siblings [4]. Their findings are consistent with our study.

Audiological complications were associated with lower HRQoL, particularly with decreased physical functioning, general health, vitality, and social functioning. Physical functioning reflects limitations in physical activities, such as difficulties walking a mile or exercising vigorously due to health problems [22]. In a SCCSS study, physical well-being was lower among younger CCS with hearing loss than for CCS with normal hearing [12]. General health reflects current and future health perceptions; for example, how people perceive their health when compared with peers or whether their health deteriorates in the future [22]. General health was also heavily impaired among CCS when compared with siblings or the general population in previous studies [25, 29]. The SF-36 assesses vitality with questions such as whether people feel full of energy or tired and worn out [22]. Previous studies of the general population showed—depending on severity—patients with tinnitus experience comorbidities, such as sleep disturbance, fatigue, and depression [10, 11, 42]. There can be bi-directional effects and vicious circles, as people with e.g. anxiety disorder can experience tinnitus as more severe. This might have been reflected in our study by the observed association with role emotional [11, 43]. Hearing loss possibly leads to feelings of fatigue from long periods of effortful listening [44–46]. Impaired social functioning refers to limitations in social activities, such as visiting family and friends, due to physical or emotional health problems [22]. CCS with hearing loss reported psychosocial difficulties and communication problems in previous studies examining the impact of hearing loss on HRQoL [12–14]. Yet, a direct comparison with our study remains difficult because they only included children and adolescents—a study population whose social behavior differs from our adult study population (median age 31 at survey). Data from adult CCS participating in the St. Jude Lifetime Cohort Study showed treatment-related hearing loss associated with reduced social attainment, which possibly relates to decreased social engagement [47]. However, none of these studies investigated the impact of tinnitus on social behavior and attainment.

### Potential causality between auditory complications and HRQoL

The observed association of lower vitality and social functioning possibly relates to educational and psychosocial problems caused by auditory complications [8, 10, 11, 13]. Other chronic health problems, such as musculoskeletal or neurological, also affect HRQoL [29]. In our study, we could not adjust for other chronic health problems. Since the risk of auditory complications and other chronic health problems increases with more intensive cancer treatment, unobserved late effects in other organ systems could contribute to lower physical and mental HRQoL (Supplement Fig. S1) [4, 5, 7, 48, 49]. However, we observed hearing loss with additional tinnitus reduces HRQoL more than hearing loss alone. Since we assumed the burden on daily life is greater when CCS experience both hearing loss and tinnitus, it possibly indicates a causal relationship [34]. Interestingly, tinnitus alone also had a greater impact on HRQoL than hearing loss alone. Since data are self-reported and tinnitus is probably underreported in our study, further research using objective hearing tests and validated instruments to assess tinnitus are important to understand its impact on CCS [33, 50].

### Conclusion

Our study showed that hearing loss and tinnitus are associated with reduced HRQoL among CCS— particularly among survivors with both, hearing loss and tinnitus. Our findings support current guideline recommendations for timely referrals to audiologists for tinnitus symptoms and optimized treatment of hearing loss and tinnitus [33]. In addition to treatment for hearing loss, there are also several treatment options for tinnitus that can benefit affected CCS [15, 50–52]. To further elaborate on causality and gain a better understanding of which aspects of quality of life are affected by auditory complications in CCS, we suggest that future studies use a quality of life questionnaire specifically designed for auditory impairments [53–55].

## Supplementary information

Below is the link to the electronic supplementary material.

ESM 1 (DOCX 151 KB)

## Data Availability

The data that support the information of this manuscript were accessed on secured servers of the Institute of Social and Preventive Medicine at the University of Bern. Individual-level sensitive data can only be made available for researchers who fulfil the respective legal requirements. All data requests should be communicated to the corresponding author.

## Abbreviations

*BMI*: Body Mass Index
*BP*: Bodily pain
*CCS*: Childhood cancer survivors
*CCSS*: American Childhood Cancer Survivor Study
*CH*: Switzerland
*CI*: Confidence interval
*CNS*: Central nervous system
*Coef*.: Estimated beta coefficient from linear regression
*CRT*: Cranial radiation therapy
*CZ*: The Czech Republic
*DE*: Germany
*FR*: France
*GH*: General health
*HRQoL*: Health-related quality of life
*HSCT*: Hematopoietic stem cell transplantation
*ICCC-3*: International Classification of Childhood Cancer, Third edition
*IQR*: Interquartile range
*MCS*: Mental component summary
*MH*: Mental health
*PCL*: PanCareLIFE
*PCS*: Physical component summary
*PF*: Physical functioning
*RE*: Role emotional
*RP*: Role physical
*SCCSS*: Swiss Childhood Cancer Survivor Study
*SF*: Social functioning
*SF-36*: Short-Form 36 questionnaire
*VT*: Vitality
